# Effects of neoadjuvant chemotherapy for patients with obstructive colon cancer: A multicenter propensity score‐matched analysis (YCOG2101)

**DOI:** 10.1002/ags3.12736

**Published:** 2023-08-31

**Authors:** Kazuya Nakagawa, Atsushi Ishibe, Hiroki Ohya, Mayumi Ozawa, Yusuke Suwa, Jun Watanabe, Hirokazu Suwa, Kanechika Den, Koichi Mori, Masashi Momiyama, Koki Goto, Itaru Endo

**Affiliations:** ^1^ Department of Gastroenterological Surgery Yokohama City University Graduate School of Medicine Yokohama Japan; ^2^ Department of Surgery, Gastroenterological Center Yokohama City University Medical Center Yokohama Japan; ^3^ Department of Surgery Yokosuka Kyousai Hospital Yokosuka Japan; ^4^ Department of Surgery Yokohama City Minato Red Cross Hospital Yokohama Japan; ^5^ Department of Surgery Fujisawa City Hospital Fujisawa Japan; ^6^ Department of Surgery NTT Medical Center Tokyo Tokyo Japan; ^7^ Department of Surgery Yokohama Medical Center Yokohama Japan

**Keywords:** diverting stoma, neoadjuvant chemotherapy, obstructive colon cancer

## Abstract

**Aim:**

Obstructive colon cancer is locally advanced colon cancer with poor prognosis. However, the effect of neoadjuvant chemotherapy (NAC) on obstructive colon cancer remains unclear. Therefore, this study aimed to investigate the safety and efficacy of NAC in patients with obstructive colon cancer.

**Methods:**

From January 2012 to December 2017, we collected patient data for clinical stage II/III obstructive colon cancer at seven Yokohama Clinical Oncology Group (YCOG) institutions. The long‐term outcomes of the NAC and non‐NAC groups were analyzed retrospectively after adjusting for patients' background characteristics using propensity score matching.

**Results:**

Among the 202 eligible patients, propensity score matching extracted 51 patients each for the NAC and non‐NAC groups. After matching, the groups showed no marked differences in the background factors. All the patients in the NAC group underwent diverting stoma construction. Nineteen patients (37.3%) experienced grade 3–4 adverse events during NAC. The incidence of postoperative complications was similar between groups. The 5‐year progression‐free survival rates were 75.8% in the NAC group and 63.0% in the non‐NAC group (*p* = 0.22, log‐rank test). The 5‐year overall survival rates were 88.5% in the NAC group and 78.8% in the non‐NAC group (*p* = 0.09, log‐rank test).

**Conclusion:**

Although NAC was feasible for obstructive colon cancer after diverting stoma construction, its effects on long‐term outcomes could not be proven.

## Graphical abstract

In this multicenter cohort study adjusted using propensity score matching, we compared the short‐ and long‐term outcomes of neoadjuvant chemotherapy (NAC) to that of non‐NAC for patients with obstructive colon cancer (OCC). Although NAC for OCC after diverting stoma construction was safe and tolerable, its efficacy on the long‐term outcomes could not be proven. However, patients in the NAC group tended to have better overall survival than those in the non‐NAC group.

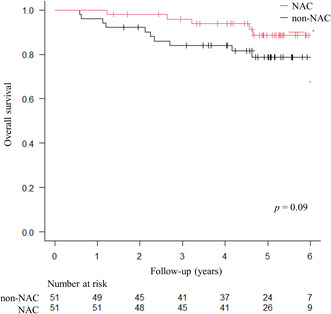

## 1 INTRODUCTION

Obstructive colon cancer (OCC) is estimated to occur in 7%–16% of colon cancer cases and often requires emergency intervention, such as emergency surgery.^1^, ^2^, ^3^, ^4^ Emergency surgery for OCC usually has higher morbidity and mortality rates and more frequently requires stoma construction than elective surgery.^5^, ^6^, ^7^ Indeed, a permanent stoma is required in up to 40% of patients and significantly reduces their quality of life.^8^, ^9^ To avoid emergency surgery and the need for a permanent stoma, we usually select long tube placement, diverting stoma construction, or self‐expandable metallic stent (SEMS) placement for bowel decompression.^10^, ^11^

Another limitation is that OCC has a poor prognosis. We previously reported that OCC has a poor prognosis, and that R1 resection and pathological T4 tumors are independent prognostic factors.^2^ Preoperative therapy, such as neoadjuvant chemotherapy (NAC), may be a good treatment option for improving the outcomes of OCC. Theoretically, NAC may improve the R0 resection rate by reducing the tumor volume and may reduce or eliminate potential lymph nodes and/or distant micrometastases via the early delivery of systemic therapy compared to adjuvant chemotherapy.^12^, ^13^ Although NAC may be a promising treatment strategy for improving the prognosis of OCC, there is insufficient evidence to prove the efficacy and safety of NAC for OCC.

Some randomized controlled trials (RCTs) have evaluated the efficacy of NAC in locally advanced colon cancer but not OCC. The FOxTROT trial demonstrated a significant decrease in the R1 resection rate and a nonsignificant trend toward better oncological outcomes at 2 years.^14^ However, only a few patients in the FOxTROT trial had colonic obstructions (2.7%). Therefore, we cannot conclude that NAC is effective for treating OCC.

Although an RCT on the efficacy of NAC for OCC has never been reported, we previously conducted a prospective, single‐arm, multicenter trial and reported good oncological outcomes.^15^ Following this trial, we concluded that NAC using mFOLFOX6 was feasible and might be a viable treatment option for patients with OCC. However, our trial did not compare NAC with upfront surgery followed by adjuvant chemotherapy.

Therefore, as an alternative to an RCT, we conducted a retrospective, multicenter study to evaluate the efficacy and safety of NAC for resectable OCC compared with upfront surgery after adjusting for patients' background characteristics using propensity score matching.

## 2 METHODS

### 2.1 Patients

This multicenter retrospective study was conducted to evaluate the safety and efficacy of NAC in patients with OCC at seven institutes participating in the Yokohama Clinical Oncology Group (YCOG) in Japan from January 2012 to December 2017. We registered the study protocol with the Ethical Advisory Committee of Yokohama City University Graduate School of Medicine and the institutional review board of each participating hospital. After receiving approval from each institutional ethics committee, patient data were collected from clinical reports (IRB number: B210100048). This study conforms to the provisions of the Declaration of Helsinki.

The eligibility criteria were as follows: (1) age ≥20 years (no upper age limit was applied); (2) OCC requiring preoperative decompression; (3) tumor located from the cecum to the rectosigmoid colon; (4) clinical stage II or III; and (5) histologically proven adenocarcinoma, signet cell carcinoma, or mucinous carcinoma. The exclusion criteria were as follows: (1) patients preoperatively diagnosed with distant metastasis and (2) patients with a history of any malignant tumors within the previous 5 years. There are no uniform criteria for the indication of NAC. Many surgeons consider patients to be eligible for NAC if they lack severe comorbidities and have a good performance status (PS). According to the surgeon's discretion and patient's choice, patients received NAC, with the regimen and number of cycles left to the surgeon's discretion. Because this study was retrospective, written informed consent was not obtained. An opt‐out was used to disclose the study information.

The primary endpoint of this study was 5‐year progression‐free survival (PFS) after adjusting for the patients' background characteristics using propensity score matching. The secondary endpoints were 5‐year overall survival (OS), all postoperative complications within 30 days [Clavien–Dindo classification (CD) grade ≥II], pathological effects of NAC, and adverse events (AEs) during NAC. Tumor regression grade (TRG) was evaluated using the Dworak TRG system,^16^ with grade characteristics as follows: grade 0, no regression; grade 1, minor regression; grade 2, moderate regression; grade 3, good regression; and grade 4, total regression. Toxicity and AEs were assessed according to the National Cancer Institute Common Toxicity Criteria (NCI‐CTC). Clinical and pathological data were retrospectively collected from the medical records. The TNM classification was recorded using the 8th TMN classification of malignant tumors. The PS was assessed using the Eastern Cooperative Oncology Group (ECOG) scale.

### 2.2 Statistical analyses

All statistical analyses were performed using the software program EZR (Saitama Medical Center, Jichi Medical University, Saitama, Japan), which is a graphical user interface for R (The R Foundation for Statistical Computing, Vienna, Austria).^17^ Case matching was performed using propensity scores calculated from the following six variables: age, sex, location (right‐sided/left‐sided), clinical T stage (T3/T4), clinical N stage (N0/N1/N2), and ECOG‐ PS (0/1/2). Nearest‐neighbor matching without replacement within a caliper was used. According to the suggestion of Austin,^18^ we set the size of the caliper as 0.2 of the standard deviation of the logit of the estimated propensity score. We excluded patients who were found outside the caliper and unmatched patients. Continuous variables were recorded as median and interquartile range (IQR) and compared using the Mann–Whitney *U* test, whereas categorical variables were recorded as frequency and proportion (%) and compared using Fisher's exact test and Pearson's chi‐squared test. Statistical significance was set at *p* values <0.05.

The PFS was calculated from the date of colonic decompression until the date of confirmed recurrence, any cause of death, or last follow‐up. OS was calculated from the date of colonic decompression until death from any cause or the date of the last follow‐up. Survival curves were estimated using the Kaplan–Meier method, and comparisons were performed using the log‐rank test.

## 3 RESULTS

### 3.1 Patients' characteristics

**FIGURE 1:**
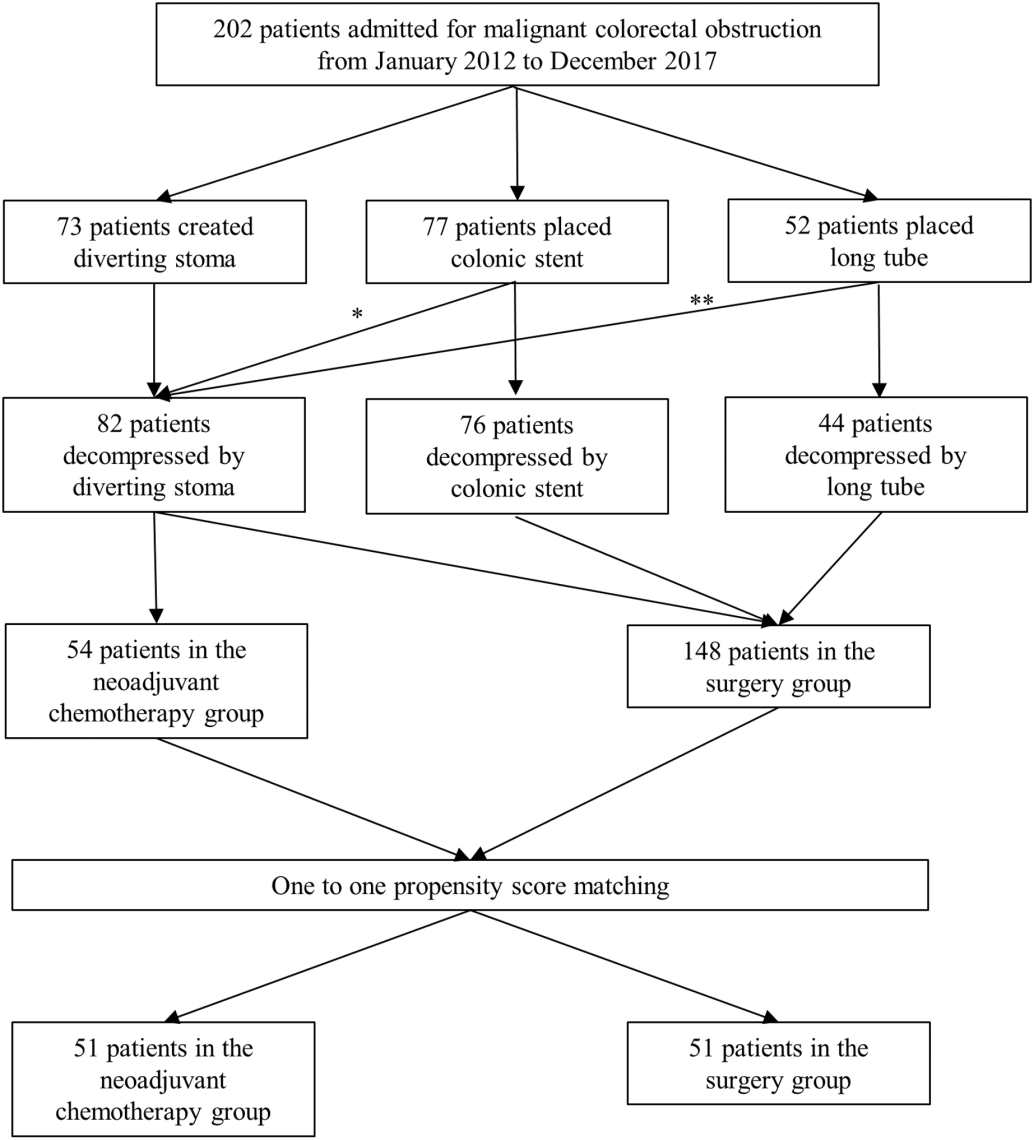
Flowchart of patient allocation. *One patient created diverting stoma for persistent obstruction; **eight patients created diverting stoma for persistent obstruction.

A patient flowchart is shown in Figure 1. In total, 202 patients were included in this retrospective study. For colonic decompression, 73 patients underwent diverting stoma construction, 77 underwent SEMS placement, and 52 underwent long tube placement (a trans‐anal tube or an intestinal long tube). One patient underwent diverting stoma construction for persistent colonic obstruction after SEMS placement, and eight patients underwent it after long tube placement. NAC was administrated to 54 patients (26.7%). Before matching, 54 and 148 patients were classified into the NAC and non‐NAC groups, respectively. Patients in the NAC group were younger, had better ECOG‐PS, and had a higher rate of clinical T4 stage disease than those in the non‐NAC group (Table 1). Therefore, case matching was performed using propensity scores calculated from the following six variables: age, sex, location (right‐sided/left‐sided), clinical T stage (T3/T4), clinical N stage (N0/N1/N2), and ECOG‐PS (0/1/2).

**TABLE 1:** Baseline patient and tumor characteristics.

Variables	Overall cohort		*p* Value	After matching		*p* Value
	NAC group (*n* = 54)	Non‐NAC group (*n* = 148)		NAC group (*n* = 51)	Non‐NAC group (*n* = 51)	
Age (years)	68 [64–74]	74 [66–80]	<0.01	69 [65–74]	68 [61–74]	0.97
Gender						
Male	30 (55.6)	91 (61.5)	0.52	27 (52.9)	26 (51.0)	1.00
Female	24 (44.4)	57 (38.5)		24 (47.1)	25 (49.0)	
ECOG performance status						
0	48 (88.8)	84 (56.7)	<0.01	45 (88.2)	46 (90.2)	1.00
1	3 (5.6)	39 (26.4)		3 (5.9)	3 (5.9)	
2	3 (5.6)	22 (14.9)		3 (5.9)	2 (3.9)	
3	0	3 (2.0)				
Location						
Right‐sided	20 (37.0)	43 (29.1)	0.31	20 (39.2)	16 (31.4)	0.54
Left‐sided	34 (63.0)	105 (70.9)		31 (60.8)	35 (68.6)	
Clinical T stage						
T3	14 (25.9)	69 (46.6)	0.01	14 (27.5)	13 (25.5)	0.51
T4a	28 (51.9)	64 (43.2)		26 (50.9)	31 (60.8)	
T4b	12 (22.2)	15 (10.1)		11 (21.6)	7 (13.7)	
Clinical N stage						
N0	19 (35.2)	57 (38.5)	0.78	17 (33.3)	20 (39.2)	0.73
N1	19 (35.2)	44 (29.7)		19 (37.3)	15 (29.4)	
N2	16 (29.6)	47 (31.8)		15 (29.4)	16 (31.4)	
Clinical stage						
IIA	11 (20.4)	36 (24.3)	0.79	11 (21.6)	8 (15.7)	0.50
IIB	6 (11.1)	19 (12.8)		4 (7.8)	10 (19.6)	
IIC	2 (3.7)	2 (1.4)		2 (3.9)	2 (3.9)	
IIIA	0	0		0	0	
IIIB	18 (33.3)	44 (29.7)		18 (35.3)	15 (29.4)	
IIIC	17 (31.5)	47 (31.8)		16 (31.4)	16 (31.4)	
Decompression method						
SEMS	0	76 (51.4)	—	0	23 (45.1)	—
Transanal tube	4 (7.4)	40 (27.0)		4 (7.8)	17 (33.3)	
Intestinal long tube	1 (1.9)	14 (9.5)		1 (2.0)	6 (11.8)	
Ileostomy	44 (81.5)	13 (8.8)		42 (82.4)	5 (9.8)	
Colostomy	10 (18.5)	15 (10.1)		9 (17.6)	7 (13.7)	
NAC regimen						
mFOLFOX6	49 (90.7)	—	—	47 (92.1)	—	—
mFOLFOX6 + anti EGFR antibody	4 (7.4)	—		3 (5.9)	—	
mFOLFOX6 + anti VEGF antibody	1 (1.9)	—		1 (2.0)	—	
Adjuvant chemotherapy	43 (79.6)	57 (38.5)	<0.01	41 (80.4)	27 (52.9)	<0.01

*Note*: Numerical data are indicated as medians. Values in parentheses are percentages, and values in brackets are the interquartile range with the first to third quartile.

Abbreviations: ECOG, Eastern Cooperative Oncology Group; EGFR, epidermal growth factor receptor; NAC, neoadjuvant chemotherapy; SEMS, self‐expandable metal stent; VEGF, vascular endothelial growth factor.

After matching, 51 patients each in the NAC and non‐NAC groups were extracted. Clinicopathological characteristics of the patients are summarized in Table 1. The median age of the patients was 69 (IQR 64–74) years in the NAC group and 68 (IQR 61–74) years in the non‐NAC group. There were no marked differences in the background characteristics after matching. In the NAC group, all the patients underwent diverting stoma construction for preoperative decompression. About half of the patients in the non‐NAC group underwent SEMS placement. Forty‐one patients (80.4%) in the NAC group and 27 (52.9%) in the non‐NAC group received adjuvant chemotherapy (AC). The rate of AC was higher in the NAC group than in the non‐NAC group (*p* < 0.01). In the NAC group, 39 patients received the mFOLFOX6 regimen for AC, two received the CAPOX regimen, and one received the UFT/LV regimen. Most patients were treated for 3 months. In the non‐NAC group, 11 patients received the CAPOX regimen, nine received the UFT/LV regimen, three received the SOX regimen, three received the capecitabine regimen, and one received the mFOLFOX6 regimen. Most patients were treated for 6 months.

### 3.2 NAC

All 51 patients in the NAC group received FOLFOX‐based chemotherapy. An anti‐epidermal growth factor receptor (EGFR) agent was combined in three patients and an anti‐vascular endothelial growth factor (VEGF) agent in one patient. Forty‐seven patients received the mFOLFOX6 regimen, three received the mFOLFOX6 plus panitumumab regimen, and one received the mFOLFOX6 plus bevacizumab regimen. Six cycles of NAC for 3 months were planned for all 51 patients and 43 (84.3%) completed NAC. As defined by RECIST ver. 1.1, a clinical complete response was observed in two patients (3.8%), a clinical partial response in 33 patients (64.7%), clinically stable disease in 15 patients (29.4%), and progressive disease in one patient (2.0%).

The details of the AEs based on the duration of NAC are summarized in Table 2. Thirty‐five patients (68.6%) had grade ≥2 AEs, and 19 (37.3%) had grade ≥3 AEs. The most common grade ≥3 AEs were neutropenia (23.5%) and anorexia (7.8%). Although nine patients (17.6%) had grade ≥2 peripheral sensory neuropathy, only one patient (2.0%) had grade ≥3 peripheral sensory neuropathy. Two patients (3.9%) had an abdominal infection (abscess) after mFOLFOX6 treatment (one and three cycles) and underwent surgery before completion of NAC.

**TABLE 2:** Neoadjuvant chemotherapy‐related adverse events.

Variables (*N* = 51)	Grade 2	Grade 3 or 4
Hematological toxicity		
Neutropenia	9 (17.6)	12 (23.5)
Leukopenia	6 (11.8)	3 (5.9)
Anemia	5 (9.8)	0
Thrombocytopenia	2 (3.9)	0
Creatinine increased	1 (2.0)	0
Hypomagnesemia	1 (2.0)	0
Non‐hematological toxicity		
Anorexia	6 (11.8)	4 (7.8)
Nausea and vomit	4 (7.8)	2 (3.9)
Abdominal infection	0	2 (3.9)
Peripheral sensory neuropathy	8 (15.7)	1 (2.0)
Fatigue	3 (5.9)	1 (2.0)
Diarrhea	2 (3.9)	1 (2.0)
Mucositis	2 (3.9)	1 (2.0)
Gastric ulcer	0	1 (2.0)
Rash acneiform	2 (3.9)	0
Dizziness	1 (2.0)	0

*Note*: Values in parentheses are percentages.

### 3.3 Surgical outcomes

Surgical outcomes are summarized in Table 3. The median time to surgery from the start of treatment was longer [127 (IQR 117–146) days vs. 17 (IQR 12–28) days; *p* < 0.01] and the proportion of patients undergoing laparoscopic surgery was higher [42/51 (82.4%) vs. 31/51 (60.8%); *p* = 0.03] in the NAC group than in the non‐NAC group. Among the patients with a preoperative diverting stoma, the proportion receiving stoma closure at primary resection was not significantly different between the groups [27/51 (52.9%) in the NAC group vs. 10/12 (83.3%) in the non‐NAC group; *p* = 0.10]. The stoma rates in the NAC and non‐NAC groups were 7.8% and 0.0%, at 1 year after surgery (*p* = 0.12).

**TABLE 3:** Surgical outcomes.

Variables	NAC group (*n* = 51)	Non‐NAC group (*n* = 51)	*p* Value
Time to surgery from the start of treatment (days)	127 [117–146]	17 [12–28]	<0.01
Duration of surgery (min)	212 [192–250]	217 [176–273]	0.94
Approach			
Open surgery	9 (17.6)	20 (39.2)	0.03
Laparoscopic surgery	42 (82.4)	31 (60.8)	
Procedure			
Ileocecal resection	4 (7.8)	1 (2.0)	—
Right hemicolectomy	16 (31.4)	15 (29.4)	
Left hemicolectomy	6 (11.8)	9 (17.6)	
Sigmoidectomy	8 (15.7)	18 (35.3)	
High anterior resection	7 (13.7)	3 (5.9)	
Low anterior resection	8 (15.7)	4 (7.8)	
Hartmann's operation	1 (2.0)	0	
Total pelvic exenteration	1 (2.0)	0	
Subtotal colectomy	0	1 (2.0)	
Combined multivisceral resection	2 (3.9)	6 (11.8)	0.13
Lymph node dissection			
D0/D1 dissection	0	3 (5.9)	0.12
D2 dissection	4 (7.8)	7 (13.7)	
D3 dissection	47 (92.2)	41 (80.4)	
Covering stoma	21 (41.2)	5 (9.8)	<0.01
Combined stoma closure	27 (52.9)	10 (83.3)^a^	0.10
Blood loss (mL)	5 (0–122)	47 (5–207.5)	0.08
Postoperative complications			
All grade	13 (25.5)	14 (27.5)	0.82
Clavien‐Dindo Grade II	11 (21.6)	12 (23.5)	0.81
Details of postoperative complications			
Superficial surgical site infection	5 (9.8)	3 (5.9)	0.46
Anastomotic leakage	3 (5.9)	1 (2.0)	0.30
Ileus	2 (3.9)	2 (3.9)	1.00
Enteritis	2 (3.9)	0	0.15
Anastomotic stenosis	0	1 (2.0)	0.31
Anastomotic bleeding	0	1 (2.0)	0.31
Urinary dysfunction	1 (2.0)	0	0.31
Pneumonia	0	2 (3.9)	0.15
Others	1 (2.0)	2 (3.9)	—
Re‐operation	1 (2.0)	1 (2.0)	1.00
Postoperative hospital stay (days)	8 [6–13]	10 [7–14]	0.16

*Note*: Numerical data are indicated as medians. Values in parentheses are percentages, and values in brackets are the interquartile range with the first to third quartile.

Abbreviation: NAC, neoadjuvant chemotherapy.

^a^ 12 patients created stoma preoperatively.

The proportion of postoperative complications did not differ significantly between the two groups. Anastomotic leakage was observed in three patients (5.9%) in the NAC group and one patient (2.0%) in the non‐NAC group (*p* = 0.30). The postoperative hospital stay was similar between the groups [8 (IQR 6–13) days in the NAC group vs. 10 (IQR 7–14) days in the non‐NAC group; *p* = 0.16].

### 3.4 Pathological findings

Pathological findings are shown in Table 4. Fifty (98.0%) and 48 (94.1%) patients underwent R0 resection in the NAC and non‐NAC groups, respectively (*p* = 0.74). The number of harvested lymph nodes in the NAC group was lower than that in the non‐NAC group [18 (IQR 12–30) vs. 28 (IQR 20–38); *p* < 0.01]. The tumor regression grades after NAC were TRG4 (0%), TRG3 (17.6%), TRG2 (33.3%), TRG1 (35.3%), TRG0 (9.8%), and not evaluable (NE) (3.9%).

**TABLE 4:** Pathological outcomes.

Variables	NAC group (*n* = 51)	Non‐NAC group (*n* = 51)	*p* Value
Pathological T stage (year)			
Tis/T1	1 (2.0)	0	0.44
T2	3 (5.9)	1 (2.0)	
T3	29 (56.8)	26 (51.0)	
T4a	13 (25.5)	14 (27.4)	
T4b	5 (9.8)	10 (19.6)	
Pathological N stage (year)			
N0	37 (72.5)	26 (51.0)	0.09
N1	10 (19.6)	18 (35.3)	
N2	4 (7.8)	7 (13.7)	
Histological type			
Well	22 (43.1)	12 (23.5)	0.01
Mod	24 (47.1)	38 (74.5)	
Por or muc	5 (9.8%)	1 (2.0)	
Lymphatic invasion	25 (49.0)	37 (72.5)	0.02
Venos invasion	28 (54.9)	39 (76.5)	0.04
Pathological stage (TNM stage), (year)			
0/I	4 (7.8)	0	0.12
IIA	23 (45.1)	13 (25.5)	
IIB	7 (13.7)	9 (17.6)	
IIC	3 (5.9)	4 (7.8)	
IIIA	0	1 (2.0)	
IIIB	8 (15.7)	15	
IIIC	6 (11.8)	8 (15.7)	
IVB	0	1 (2.0)	
Number of harvested lymph node	18 [12–30]	28 [20–38]	<0.01
R			
R0	50 (98.0)	48 (94.1)	0.74
R1	1 (2.0)	1 (2.0)	
R2	0	2 (3.9)	
Tumor regression grade			
TRG4 (complete regression)	0	—	—
TRG3 (marked regression)	9 (17.6)	—	
TRG2 (moderate regression)	17 (33.3)	—	
TRG1 (little regression)	18 (35.3)	—	
TRG0 (no regression)	5 (9.8)	—	
NE	2 (3.9)	—	

*Note*: Numerical data are indicated as medians. Values in parentheses are percentages, and values in brackets are the interquartile range with the first to third quartile.

Abbreviations: NAC, neoadjuvant chemotherapy; NE, not evaluated.

### 3.5 The PFS and OS

The median follow‐up period was 60.9 months for survivors (62.7 months in the NAC group vs. 60.2 months in the non‐NAC group; *p* = 0.40).

**FIGURE 2:**
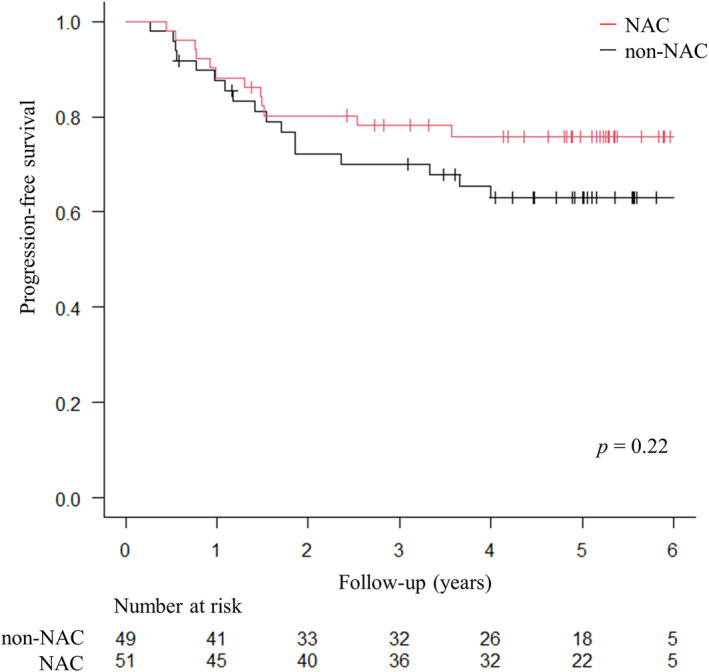
The progression‐free survival. Kaplan–Meier curve for the comparison of the progression‐free survival.

The PFS curves are shown in Figure 2. Two patients who underwent R2 resection in the non‐NAC group were excluded from the PFS analysis. During the follow‐up period, relapse occurred in 12 and 17 patients in the NAC and non‐NAC groups, respectively. The 5‐year PFS rates were 75.8% and 63.0% in the NAC and non‐NAC groups respectively, with no significant difference between the groups (*p* = 0.22, log‐rank test).

**FIGURE 3:**
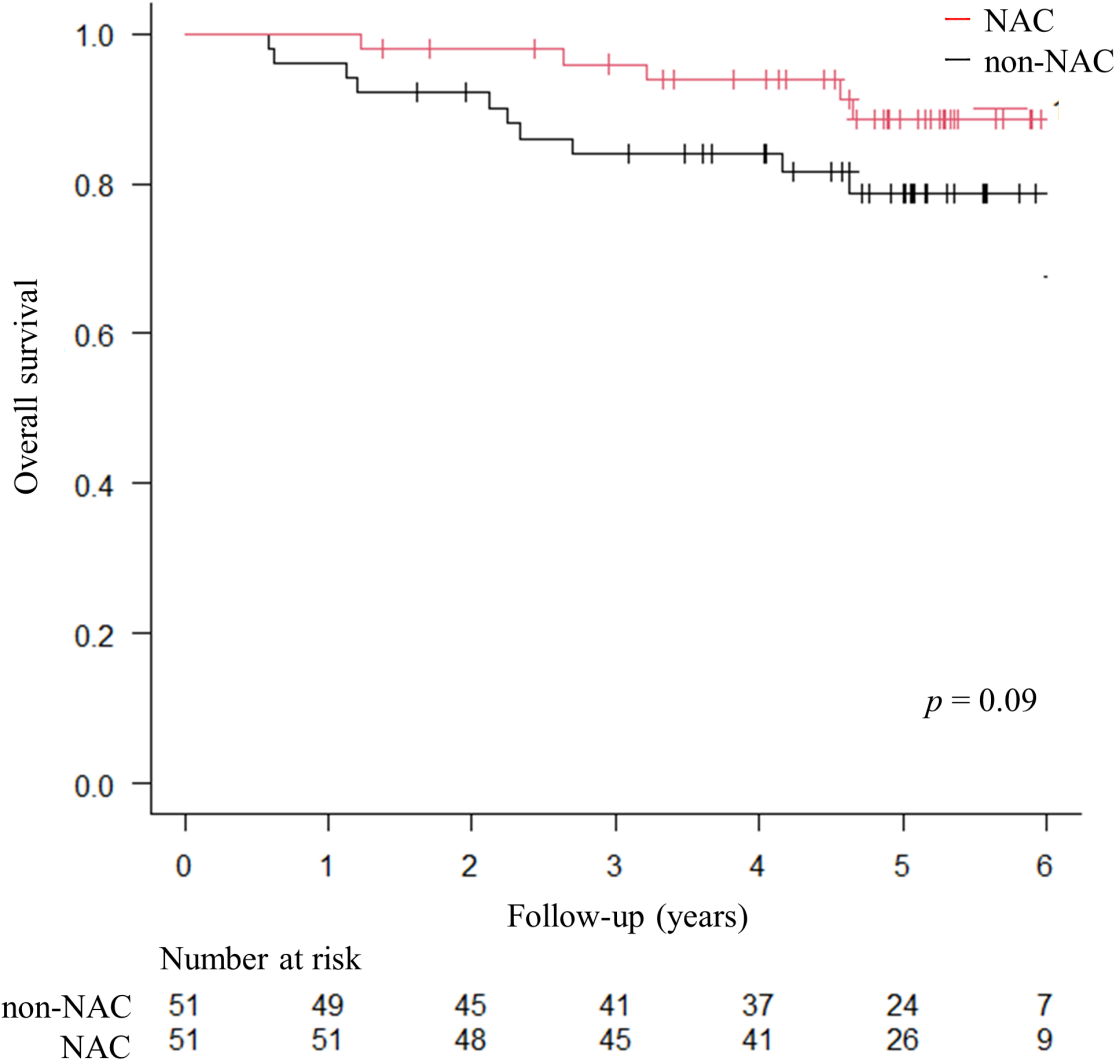
The overall survival. Kaplan–Meier curve for the comparison of the overall survival.

The OS curves are shown in Figure 3. During the follow‐up period, five patients died in the NAC group and 12 in the non‐NAC group. In the NAC group, four patients died of OCC, and one patient died of another malignant disease (gastric cancer), while in the non‐NAC group, five patients died of OCC and seven patients died of other diseases. Three patients died of other malignant diseases (pancreatic cancer, lung cancer, and diffuse large B‐cell lymphoma), two died of lung diseases (chronic obstructive pulmonary disease, and drug‐induced interstitial pneumonia), one died of heart disease (heart failure), and one died of an unknown cause. The 5‐year OS rates of the NAC and non‐NAC groups were 88.5% and 78.8%, respectively. Patients in the NAC group tended to have a better OS than those in the non‐NAC group (*p* = 0.09, log‐rank test).

## 4 DISCUSSION

In this multicenter cohort study adjusted using propensity score matching, we compared the short‐ and long‐term outcomes of NAC to those of non‐NAC in patients with OCC. Although NAC for OCC after diverting stoma construction was safe and tolerable, its efficacy on long‐term outcomes (PFS and OS) could not be proven. However, patients in the NAC group tended to have a better OS than those in the non‐NAC group. To our knowledge, this is the first study to report the long‐term outcomes of NAC after stoma construction compared with non‐NAC in patients with OCC.

Two recent studies described the safety and efficacy of NAC compared to non‐NAC, although they included only patients with left‐sided colorectal cancer obstruction.^19^, ^20^ Han et al.^19^ retrospectively compared 48 patients who underwent elective surgery after SEMS placement and NAC with 52 patients who underwent elective surgery after SEMS placement alone. They reported that laparoscopic surgery was performed more frequently and that a stoma was constructed less frequently in the NAC group than in the only‐SEMS group. Of the 48 patients in the combined treatment group, none showed complete regression, eight (16.7%) showed marked regression, 18 (37.5%) showed moderate regression, and 22 (45.8%) showed little regression according to the Dworak TRG. Thus, the authors concluded that elective surgery following SEMS placement and NAC is a safe, effective, and well‐tolerated treatment approach.

Consistent with their report, the proportion of patients who underwent laparoscopic surgery in the NAC group was higher than that in the non‐NAC group in the present study. All patients in the NAC group underwent diverting stoma construction prior to NAC. Therefore, the rate of stoma construction was not lower in the NAC group than in the non‐NAC group, in contrast to the report by Han et al. One year after primary resection, most of the diverting stomas were closed, and the stoma rate was 7.8% (4/51) in the NAC group in the present study. The proportions of patients with complications due to SEMS was 14.6% (7/48) in the NAC group and 3.8% (2/52) in the only‐SEMS group in a study by Han et al. Although there was no significant difference between the two groups, physicians should be alert to complications, such as perforation, stent migration, and re‐obstruction during NAC. Evidence concerning the safety of NAC after SEMS placement is still insufficient, and RCTs to explore these issues are needed in the future.

Zhang et al.^20^ retrospectively compared 32 patients who underwent elective surgery following NAC with 64 patients who underwent elective surgery for acute left‐sided malignant colonic obstruction using propensity score matching. Of the 32 patients who underwent NAC, 23 underwent SEMS placement, while nine underwent diverting stoma construction for colonic decompression before NAC. No complications due to SEMS occurred during the NAC. The authors reported that the stoma rate in the NAC group was significantly lower than that in the surgery group 1 year after surgery (9.4% vs. 17.7%, *p* = 0.047). They concluded that elective surgery following NAC was safe and associated with a number of advantages, such as a lower stoma rate 1 year after surgery, fewer postoperative complications, and shorter postoperative hospital stay than upfront surgery. The total number of postoperative complications was higher in the surgery group than in the NAC group (27.0% vs. 0.0%, *p* = 0.001). However, in contrast to their study, the proportion of patients with postoperative complications was not significantly different between the two groups in the present study.

The number of harvested lymph nodes was significantly smaller in the NAC group than in the non‐NAC group. We do not believe that this difference affected the prognosis, as there was no significant difference in the extent of lymphadenectomy. The time to surgery from the start of decompression was longer in the NAC group than in the non‐NAC group. It is possible that the lymph nodes that were swollen due to an inflammatory reaction shrunk and became invisible. Another possibility is that the metastatic lymph nodes became invisible due to the effects of NAC.

Generally, NAC has several advantages. We expect NAC to improve the R0 resection rate by reducing the tumor size. In the present study, 50 (98.0%) and 48 (94.1%) patients underwent R0 resection in the NAC and non‐NAC groups, respectively, with no significant differences between the groups. Because the R0 resection rate in the non‐NAC group was higher than that reported in previous studies, it is possible that NAC did not improve the R0 resection rate. We also expect NAC to eradicate micrometastatic disease via the early delivery of systemic therapy compared to AC.^12^, ^13^ Postoperative complications may delay or prevent the use of AC. In the present study, patients in the non‐NAC group had a significantly lower rate of AC than those in the NAC group, although there was no significant difference in postoperative complications. In contrast to our findings, however, there was also a report that NAC reduced postoperative complications compared with non‐NAC. Therefore, NAC can maintain good dose intensity and may lead to improved long‐term outcomes. However, the efficacy of NAC in terms of long‐term outcomes (specifically the PFS and OS) was not proven in our study.

AC is recommended to reduce the risk of recurrence and to improve the prognosis of patients with high‐risk stage II or III colon cancer. We performed an additional analysis of the effect of AC on the PFS and OS. AC was performed in 41 patients (80.4%) in the NAC group and in 27 patients (52.9%) in the non‐NAC group. In the NAC group, the 5‐year PFS rates were 80.2% and 55.6% in the AC and non‐AC groups, respectively. In the non‐NAC group, the 5‐year PFS rates were 57.8% and 68.5% in the AC and non‐AC groups, respectively. There were no significant differences among the four groups (*p* = 0.18, log‐rank test) (Figure S1). Furthermore, in the NAC group, the 5‐year OS rates were 91.6% and 71.1% in the AC and non‐AC groups, respectively. In the non‐NAC group, 5‐year OS rates were 76.3% and 85.6% in the AC and non‐AC groups. There were no significant differences among the four groups (*p* = 0.25, log‐rank test) (Figure S2).

We strongly expect that NAC will improve the prognosis of OCC. Based on the results of this study, we cannot conclude that NAC was ineffective. In our study, the 5‐year PFS rates were 75.8% and 63.0% in the NAC and non‐NAC groups, respectively, with no significant difference between the groups. When the 5‐year PFS rate improved by 12% from 63% to 75%, the α error was set to 0.05. When performing a two‐sided test (calculated by post‐hoc power analysis), the power was 0.30. If the β‐power when performing a two‐sided test was 0.80, the calculated sample size would have been 191 in each group. Therefore, the β‐power used in this study was considered insufficient. Han et al.^19^ reported the possibility of improving the long‐term outcomes of NAC in obstructive left‐sided CRC. Patients in the SEMS‐NAC group had a better OS than those in the SEMS group (53 vs. 47 months, *p* = 0.02, log‐rank test). Zhang et al.^20^ reported no significant difference in the 1‐year relapse‐free survival between the two groups (91.3% in the surgery group vs. 96.8% in the NAC group; *p* = 0.562) for obstructive left‐sided CRC. Because the efficacy of NAC has not been sufficiently proven to date and may not have been demonstrated statistically owing to insufficient power in this study, we cannot conclude that NAC is ineffective for OCC.

Currently, evidence concerning the optimal decompression method before NAC in patients with OCC is insufficient. Trans‐anal decompression tubes and nasal intestinal long tubes are not suitable for long‐term placement, because patients with OCC have difficulty with oral intake during tube placement. SEMS placement may be selected for obstructive decompression when planning a bridge to surgery without NAC. However, when planning primary resection after NAC, it is necessary to consider whether to place an SEMS or to construct a diverting stoma. Although there have been a few recent reports on the safety of NAC after SEMS placement, no recommendations were included in the 2020 European Society of Gastrointestinal Endoscopy (ESGE) guidelines, as there were no studies concerning NAC after SEMS placement in the curative setting.^21^ Furthermore, the ESGE suggests that a diverting stoma is a valid option if the patient is not a candidate for colonic stenting (e.g., locally advanced colorectal cancer that requires induction therapy). The 2019 Japan Society for Cancer of the Colon and Rectum (JSCCR) guidelines also do not recommend systemic chemotherapy after SEMS placement because “Stent treatment is not recommended for patients who are indicated for systemic therapy.”^22^ The guidelines state that this recommendation is based on the possibility of chemotherapy causing tumor shrinkage and tissue necrosis, which can lead to perforation and penetration of the surrounding organs. Based on the present study findings, we believe that NAC after constructing a diverting stoma is safe, but stoma construction has the disadvantage of lowering the quality of life. The optimal decompression method before NAC remains to be determined in the future, and we expect a well‐designed RCT to help clarify this point.

In the present study, none of the patients in the NAC group had SEMS placed, although about half of those in the non‐NAC group had one. The difference in the proportion of SEMS placement may have influenced the long‐term outcomes. SEMS placement is associated with the risk of metastasis promotion and seeding recurrence with perforation, which can worsen the prognosis.^23^ Sabbagh et al. retrospectively compared the long‐term outcomes between the SEMS and surgery‐only groups using propensity score matching. They reported that 48 patients in the SEMS group had significantly poorer 5‐year OSs than the 39 patients in the surgery‐only group. Their results suggest a worse OS of patients with left‐sided malignant colonic obstruction with SEMS insertion than of those with immediate surgery. However, a meta‐analysis that included seven studies with 1333 patients reported no significant difference in the recurrence or 3‐ or 5‐year mortality rates between the BTS and emergency surgery groups.^24^ Therefore, the impact of SEMS placement on long‐term outcomes remains controversial.

Several limitations of the present study warrant mention. First, it was retrospective in nature. As selection bias was inevitable, propensity score matching was performed. However, this approach involves worse control of clinical variables than an RCT. Second, this study was not an RCT and a standardized NAC protocol was not defined. Individual surgeons selected the NAC regimen and determined the number of cycles to administer; therefore, a selection bias was inevitable. Third, the proportion receiving AC was lower in the non‐NAC group than in the NAC group. AC is known to affect the survival and recurrence rates in patients with colon cancer. Although the difference in the proportion of AC might have influenced the long‐term outcomes, this might have been due to the influence of therapeutic intervention. Because patients who underwent NAC might have better chemotherapy compliance than those who did not undergo NAC, more patients in the NAC group might have been selected to receive adjuvant chemotherapy. Fourth, the small sample size may have led to insufficient analytical power. As discussed above, we cannot conclude that NAC is ineffective for OCC based on the results of this study. Finally, no data were available concerning microsatellite instability or mismatch repair defects in patients, which are closely associated with response to chemotherapy.

In conclusion, although NAC for OCC after diverting stoma construction is safe and tolerable, its long‐term efficacy (PFS and OS) could not be confirmed. In the future, a well‐designed RCT should be conducted.

## AUTHOR CONTRIBUTIONS

Kazuya Nakagawa and Atsushi Ishibe drafted and revised the manuscript, contributed to the study conception and design, performed the statistical analysis and data interpretation, and accepted responsibility for the conduct of research, final approval, and study supervision. Jun Watanabe revised the manuscript, contributed to the study concept and design, and performed the statistical analysis and data interpretation. Hiroki Ohya, Mayumi Ozawa, Yusuke Suwa, Hirokazu Suwa, Kanechika Den, Koichi Mori, Masashi Momiyama, and Koki Goto collected and interpreted the data, and revised the manuscript. Itaru Endo provided critical advice on drafting the manuscript. All the authors have read and approved the final manuscript.

## FUNDING INFORMATION

This study was supported by the Yokohama Clinical Oncology Group (YCOG). The YCOG is a non‐profit organization, funded by corporate and individual donations.

## CONFLICT OF INTEREST STATEMENT

The authors declare no conflicts of interest for this article. Itaru Endo and Jun Watanabe are editorial board members of the *Annals of Gastroenterological Surgery*.

## ETHICS STATEMENT

Approval of the research protocol: The study protocol was approved by the Ethical Advisory Committee of Yokohama City University Graduate School of Medicine (IRB number: B210100048) and the Institutional Review Board of each participating hospital.

Informed Consent: N/A.

Registry and Registration No. of the study/trial: Not applicable (registration was not required owing to the retrospective nature of the study).

Animal Studies: N/A.

## Supporting information


Figure S1.

Figure S2.
