# Supplementary figures and images for: Effects of neoadjuvant chemotherapy for patients with obstructive colon cancer: A multicenter propensity score‐matched analysis (YCOG2101)
Source: Ann Gastroenterol Surg. 2023 Aug 31;8(2):262–72. doi: 10.1002/ags3.12736 (PMC10914701; doi:10.1002/ags3.12736)

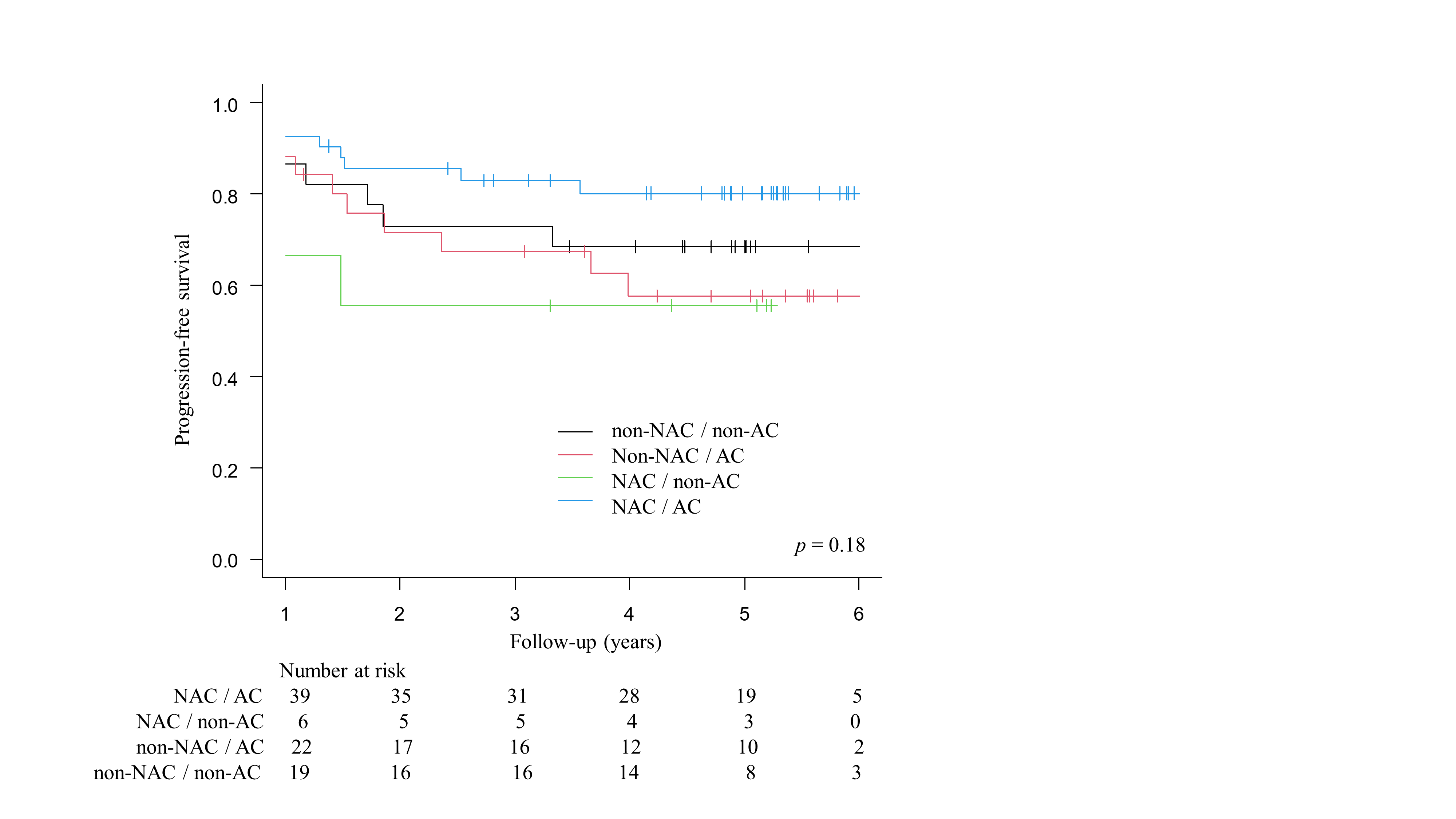

Supplement: Supplementary file 1 — Figure S1. [file AGS3-8-262-s002.tif]

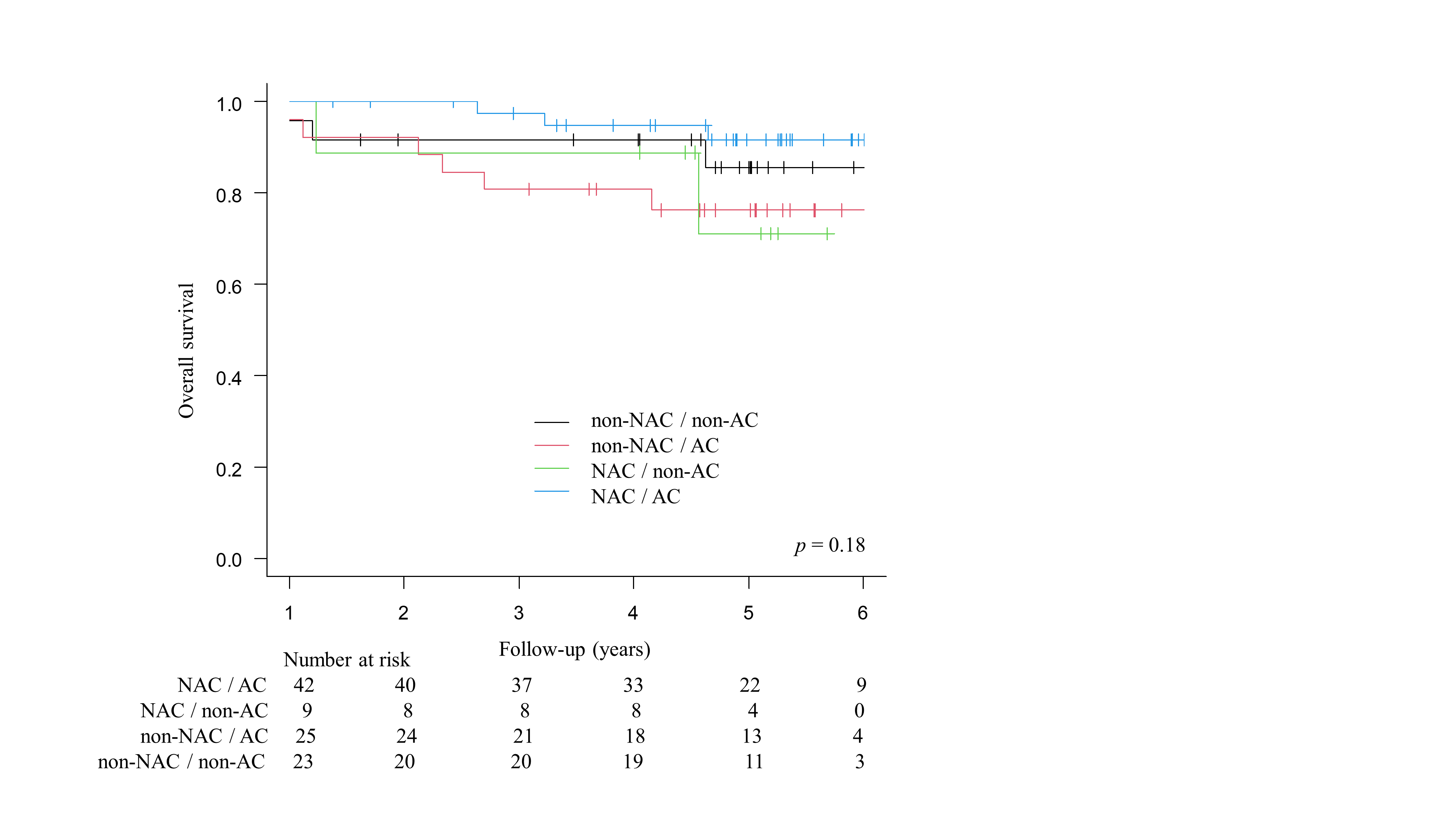

Supplement: Supplementary file 2 — Figure S2. [file AGS3-8-262-s001.tif]
